# NEU1 Regulates Mitochondrial Energy Metabolism and Oxidative Stress Post-myocardial Infarction in Mice *via* the SIRT1/PGC-1 Alpha Axis

**DOI:** 10.3389/fcvm.2022.821317

**Published:** 2022-04-25

**Authors:** Zhen Guo, Di Fan, Fang-Yuan Liu, Shu-Qing Ma, Peng An, Dan Yang, Min-Yu Wang, Zheng Yang, Qi-Zhu Tang

**Affiliations:** ^1^Department of Cardiology, Renmin Hospital of Wuhan University, Wuhan, China; ^2^Hubei Key Laboratory of Metabolic and Chronic Diseases, Wuhan, China

**Keywords:** neuraminidase 1, myocardial infarction, mitochondrial metabolism, SIRT1, oxidative stress

## Abstract

**Objective:**

Neuraminidase 1 (NEU1) participates in the response to multiple receptor signals and regulates various cellular metabolic behaviors. Importantly, it is closely related to the occurrence and progression of cardiovascular diseases. Because ischemic heart disease is often accompanied by impaired mitochondrial energy metabolism and oxidative stress. The purpose of this study was to investigate the functions and possible mechanisms of NEU1 in myocardial remodeling and mitochondrial metabolism induced by myocardial infarction (MI).

**Methods:**

In this study, the MI-induced mouse mode, hypoxia-treated H9C2 cells model, and hypoxia-treated neonatal rat cardiomyocytes (NRCMs) model were constructed. Echocardiography and histological analysis were adopted to evaluate the morphology and function of the heart at the whole heart level. Western blot was adopted to determine the related expression level of signaling pathway proteins and mitochondria. Mitochondrial energy metabolism and oxidative stress were detected by various testing kits.

**Results:**

Neuraminidase 1 was markedly upregulated in MI cardiac tissue. Cardiomyocyte-specific NEU1 deficiency restored cardiac function, cardiac hypertrophy, and myocardial interstitial fibrosis. What is more, cardiomyocyte-specific NEU1 deficiency inhibited mitochondrial dysfunction and oxidative stress induced by MI. Further experiments found that the sirtuin-1/peroxisome proliferator-activated receptor γ coactivator α (SIRT1/PGC-1α) protein level in MI myocardium was down-regulated, which was closely related to the above-mentioned mitochondrial changes. Cardiomyocyte-specific NEU1 deficiency increased the expression of SIRT1, PGC-1α, and mitochondrial transcription factor A (TFAM); which improved mitochondrial metabolism and oxidative stress. Inhibition of SIRT1 activity or PGC-1α activity eliminated the beneficial effects of cardiomyocyte-specific NEU1 deficiency. PGC-1α knockout mice experiments verified that NEU1 inhibition restored cardiac function induced by MI through SIRT1/PGC-1α signaling pathway.

**Conclusion:**

Cardiomyocyte-specific NEU1 deficiency can alleviate MI-induced myocardial remodeling, oxidative stress, and mitochondrial energy metabolism disorder. In terms of mechanism, the specific deletion of NEU1 may play a role by enhancing the SIRT1/PGC-1α signaling pathway. Therefore, cardiomyocyte-specific NEU1 may provide an alternative treatment strategy for heart failure post-MI.

## Introduction

Heart failure (HF) is the terminal stage of many cardiovascular diseases (myocardial infarction (MI), arrhythmia), with high morbidity and mortality worldwide, and is a major challenge to human health (1). Despite various treatment strategies such as drug therapy (2), left ventricular assist device (3), artificial heart (4), and heart transplantation (5), the progression of heart failure after MI is still fundamentally irreversible. Among the plethora of mechanisms related to the onset and progression of MI, mitochondria are the most concerned (6–8). Due to the heart's high demand for energy, mitochondria are highly enriched in myocardial tissue, which is essential for the function of the heart (9). Mitochondria plays an important role in ATP production, reactive oxygen formation, cell apoptosis, and signal transduction in the metabolic process of cardiomyocytes. The generation of mitochondrial reactive oxygen species (ROS) is an important early driver of MI damage, but it has been considered to be a non-specific result of the interaction between respiratory chain dysfunction and oxygen in MI (10). In fact, mitochondrial dysfunction is an important predictor of cell death (11). Thus, the involvement of mitochondrial in MI is complex, and a detailed understanding of the pathogenic and reparatory mechanisms triggered by mitochondrial mediators is a prerequisite for the development of mitochondrial-targeted therapies.

Peroxisome proliferator-activated receptor γ coactivator-1α (PGC-1α) is a key booster of mitochondrial function and metabolism. PGC-1α is highly expressed in tissues with high-energy demands (such as heart, and adipose tissue), and regulates downstream signal pathways and mitochondrial oxidative stress-related proteins (such as components of the electron transport system) (12). PGC-1α is abnormally expressed in cardiac hypertrophy, heart failure, chronic cardiomyopathy, etc. (13). The deficiency of PGC-1α promotes mitochondrial dysfunction in the progression of various diseases (14). Mammalian sirtuin 1 (SIRT1) belongs to the sirtuin protein family. It is an enzyme responsible for protein deacetylation in cell regulation. It is closely related to the pathophysiological process of inflammation, mitochondrial biogenesis, cell aging, and subsequent aging (15). Under nutrient-restricted conditions, SIRT1 regulates PGC-1α target genes and is essential in dealing with increased fatty acid oxidation (16). The deficiency of SIRT1 promotes mitochondrial dysfunction and causes dilated cardiomyopathy in mice (17).

The properties of new signal mediators related to MI and mitochondrial function are the key focus for exploring new drug targets to improve clinical outcomes. Neuraminidases (NEUs), also known as sialidase, is a family of enzymes that decompose sialic acid on the cell surface. Four mammalian NEU species (NEU1, NEU2, NEU3, and NEU4) have been identified based on differences in subcellular localization and enzyme properties, of which NEU1 is the most abundant. NEU1 has a catabolic function and participates in the structural and functional regulation of cell receptors (18). Recent research found that neuraminidase 1 (NEU1) colocalizes with some but not all mitochondria within platelets (19). Studies have shown that NEU1 upregulation in infiltrating cardiac monocytes and macrophages leads to heart failure after ischemia-reperfusion by promoting inflammation (20). In addition, plasma neuraminidase activity increased after MI compared with healthy controls (21). NEU1 is a key driver of myocardial hypertrophy, and anti-influenza drugs zanamivir and oseltamivir (viral NEU inhibitors) can significantly alleviate myocardial hypertrophy (22). However, the role of NEU1 in mitochondrial dysfunction in MI remains unknown. Based on limited clues, we hypothesized that NEU1 is involved in mitochondrial function and metabolic changes in MI.

Therefore, this study aims to determine (i) the specific effects (if any) of NEU1 on cardiac function, mitochondrial function, and metabolism post-MI; (ii) what is the mechanism of NEU1 on the changes in cardiac mitochondrial energy metabolism?

## Methods

### Animals Experimental Model

All animal management is based on the “Guidelines for the Care and Use of Laboratory Animals” published by the National Institutes of Health (NIH publication number 85–23, revised in 2001), and is supported by the Animal Care and Use Committee of Renmin Hospital of Wuhan University. C57/BL6 mice (8–10 weeks) were purchased from the Institute of Laboratory Animal Science (Beijing, China). After 7 days of adaptation, we use continuous inhalation of 1.5% isoflurane to anesthetize mice, and MI was established as described previous (23). Simply put in order to accurately expose the heart, a left thoracic incision was made in the fifth intercostal space. Permanently ligate the anterior descending branch of the left coronary artery with 6-0 silk thread. In total 4-0 nylon thread is used to suture the skin after ligation, except for ligating the LAD coronary arteries, sham-operated mice used the same procedure. Place these mice in a 37°C constant temperature cage until they recover. According to the manufacturer's manual, 4 weeks before LAD ligation, the adeno-associated virus 9 (AAV9)-targeted to NEU1 and injected directly into the myocardium at three different locations in the ischemic area. The dose was 1 × 10^10^ VP (24). Mice were randomized into four groups: Sham + AAV9-shRNA, Sham + AAV9-shNEU1, MI + AAV9-shRNA, and MI + AAV9-shNEU1 groups.

Conditional PGC-1α deletion was generated by crossing PGC-1α^flox/flox^ mice (Cyagen, China) with mice carrying α-MHC-MEM-Cre transgene (Jackson Laboratory, USA). For cardiac specific-knockout of PGC-1α, pharmaceutical-grade tamoxifen dissolved in corn oil was injected into 6-week old mice for five consecutive days (50 mg/kg per day, i.p.). The PCR method was used to identify the genomic DNA of the tail of PGC-1α-cKO mice. Perform functional data and gene expression level analysis in pairs of male a-MHC-PGC-1α-KO (cKO) and male littermates (Cre), PGC-1α knockout male mice (8–10 weeks old) that weighed between 22 and 27g were used in all experiments and maintained a 12/12-h light–dark cycle in a temperature and humidity-controlled room.

### Echocardiography

All the mice were anesthetized with 1.5% isoflurane and placed supine on a heating pad (37°C). The echocardiography was performed with MyLab 30CV ultrasound (Biosound Esaote Inc.) with a 10-MHz linear array ultrasound transducer to evaluate the cardiac function of mice after MI or sham treatment (25).

### Western Blot

Heart tissues and cultured cardiomyocytes were collected and lysed by RIPA lysis buffer. In a subset of the experiment, the previously described method was used to separate mitochondria and cytoplasmic components in heart tissue samples (26). The same amount of protein was transferred to the PVDF membrane after electrophoresis. The membrane was blocked, and the primary antibody was incubated overnight under 4°C. The primary antibodies used are listed as follows: NEU1(#ab233119; 1:1000, Abcam); Mitofusin 2 (MFN2) (#9482S; 1:1,000, CST); dynamin-related protein 1 (DRP1) (# SC-32898, 1:500, SANTA); SIRT1 (# ab110304, 1:1000, Abcam); PGC-1alpha (# ab191838, 1:1,000, Abcam); transcription factor A, mitochondrial (TFAM) (#7495, 1:1,000, CST); voltage-dependent anion channels (VDAC) (#ab191440, 1:1,000, Abcam);. GAPDH (#2722, 1:1,000, CST). Then incubated with the secondary antibody for 1 h at room temperature. The protein bands were observed using ChemiDoc XRS (Bio-Rad Laboratories, Inc.)+ system with ECL reagents, and the intensity of bands was quantified by using Image J, and the protein abundance was normalized to the levels of GAPDH or VDAC.

### Real-Time PCR

Extract total RNA from mouse myocardial tissue or H9C2 cells with TRIzol reagent (Roche, 11667165001). Use 2 μg total RNA and transcript First Stand cDNA Synthesis Kit (Roche, 04897030001) for cDNA synthesis reaction. The LightCycler 480 real-time PCR system (Roche) is used to perform a real-time quantitative PCR reaction with a volume of 20 μl. The gene expression level was normalized to the GAPDH gene expression level, and the relative mRNA level was quantified with the internal control. The primers used are presented as follows: Mice-TGFβ1: 5′-ATCCTGTCCAAACTAAGGCTCG-3′(F), 5′-ACCTCTTTAGCATAGTAGTCCGC-3′(R); Mice-Col1: 5′-CCCAACCCAGAGATCCCATT-3′(F), 5′-GAAGCACAGGAGCAGGTGTAGA-3′(R); Mice-Col3: 5′- GATCAGGCCAGTGGAAATGT-3′(F), 5′-GTGTGTTTCGTGCAACCATC-3′(R);

Mice-GAPDH: 5′- TCATCAACGGGAAGCCCATC-3′(F), 5′- CTCGTGGTTCACACCCATCA-3′(R).

### Histological Analysis

Take out the heart and immediately place it in 10% potassium chloride solution, squeeze out the blood in the heart cavity, and place it in 10% formalin. The hearts were dissected into 5 μm slices. Wheat germ agglutinin (WGA) for histopathology to determine the myocyte cross-sectional area (CSA). Picro-Sirius red (PSR) for determining cardiac fibrosis.

### Mitochondrial Isolation and Mitochondrial Respiratory Chain Complex Activity

According to the manufacturer's instruction, heart mitochondria was isolated by Tissue Mitochondria Isolation Kit (C3606, Beyotime). The specific activity of complex I (27), complex II (28), complex III (29), and complex IV (30) in myocardial tissues was determined by the methods previously contributed by investigators.

### Analysis of Mitochondrial ROS

The MnSOD Assay Kit with WST-8 (Beyotime, S0103) was used to detect the MnSOD activities of fresh heart or NRCMs according to the manufacturer's instructions.

Stain NRCMs with mitochondria-specific superoxide indicator triphenylphosphonium-linked hydroethidium (MitoSOX) (M36008, Thermo Fisher Scientific) to detect the production of mitochondrial superoxide. The mitochondrial subcellular location of MitoSOX was confirmed by co-labeling with 50 nM MitoTracker Green (C1048, Beyotime). ROS production in the fresh heart was assessed by dihydroethidium (DHE) staining (31). A fluorescence microscope (OLYMPUS DX51) and DP2-BSW software (version 2.2) were used to obtain the representative images.

### ATP Content

According to the manufacturer's instruction, an ATP content kit (S0026, Beyotime) was used to detect cellular ATP content. ATP level was further normalized to protein content.

### Quantification of mtDNA

According to the manufacturer's instructions, use the DNeasy Blood and Tissue Kit (Qiagen, 69504) to collect heart tissue or cells to isolate total DNA. Mitochondrial DNA (mtDNA) was quantified by mitochondrial NADH-ubiquinone oxidoreductase chain 1 (MT-ND1) and nuclear DNA (nDNA) named GAPDH. Mouse primers are presented as follows: MT-ND1: 5′-TCTAATCGCCATAGCCTTCC-3′(F), 5′-GCGTCTGCAAATGGTTGTAA-3′(R); GAPDH: 5′-GTCAAGGCAGAGAACGGGAA-3′(F), 5′-GGTTCACGCCCATCACAAAC-3′(R).

### Cell Culture and Treatments

To examine the levels of mitochondrial biogenesis and function *in vitro*, neonatal rat cardiac myocytes (NRCMs) were isolated and cultured in Dulbecco's modified Eagle's/F-12 (11330, Gibco, Grand Island, NY, USA), supplemented with 15% fetal bovine serum (10099, Gibco) as previously described (25). We ordered H9C2 cells from the Cell Bank of the Chinese Academy of Sciences (Shanghai, China) and cultured them in DMEM containing 10% fetal bovine serum and 1% penicillin-streptomycin. There was no mycoplasma contamination in the cells. This study did not use common misidentified cells.

Adenoviral vectors carrying NEU1 small hairpin RNAs (shNEU1) or the scrambled shRNA were used to infect NRCMs or H9C2 cells at an MOI of 100 for 4 h to produce transfected cells with stable NEU1 knockdown expression. NRCMs or H9C2 cells are hypoxic at 1% oxygen concentration to simulate MI injury *in vivo*. To examine the functional role of SIRT1 or PGC-1α *in vitro*, NRCMs or H9C2 cells were treated with SIRT1 inhibitor EX5727 (40 μM, HY-15452, MCE) (32) or PGC-1α inhibitor SR-18292 (10 μM; HY-101491, MCE) (33) was added to the cell medium to determine its inhibitory effects on mitochondrial.

### Statistical Analysis

The data were expressed as mean ± SEM. Survival data were analyzed by the Kaplan–Meier method followed by a Mantel–Cox log rank test. Student's unpaired *t*-test was used to compare the two groups. One-way ANOVA followed by Tukey's *post hoc* test was used among multiple groups. *P* < 0.05 indicates that the difference is statistically significant.

## Results

### NEU1 Expression Was Increased in MI Tissues

**Figure 1:**
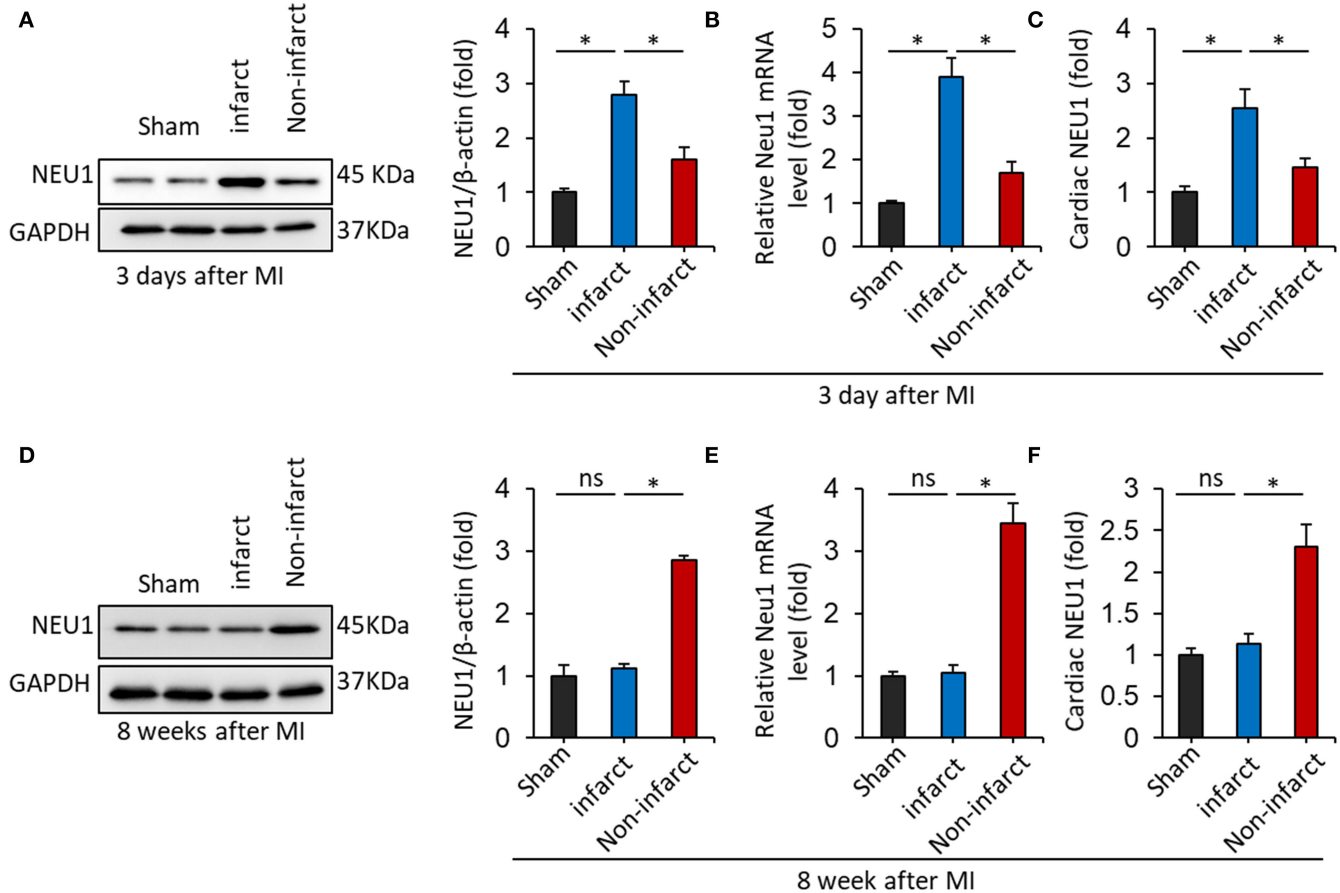
NEU1 expression was increased in mice heart post-MI. **(A)** Immunoblot analysis of NEU1 protein (*n* = 6) in the mouse heart 3 days after LAD surgery. **(B)** RT-PCR analysis of NEU1 mRNA in the mouse heart 3 days after MI. **(C)** Cardiac NEU1 expression detected by ELISA (*n* = 6). **(D)** Immunoblot analysis of NEU1 protein (*n* = 6) in the mouse heart 28 days after LAD surgery. **(E)** RT-PCR analysis of NEU1 mRNA in the mouse heart 28 days after MI. **(F)** Cardiac NEU1 expression detected by ELISA (*n* = 6). *p < 0.05 vs. corresponding group. n.s., non-significant.

First of all, immunoblot showed dramatically elevated NEU1 expression in the mice hearts 3 days after MI, compared with sham-operated controls. Moreover, the NEU1 protein level in infarct area of mouse hearts 3 days after MI increased greater than in the non-infarct area (Figure 1A). Similar results were obtained in regard to the effect of MI on mRNA expression of NEU1 (Figure 1B). ELISA data further confirmed that MI stimulation increased NEU1 expression in mice hearts (Figure 1C). Our finding of elevated NEU1 expression after acute MI model is consistent with previous observations (34). Next, we further investigated confirmed NEU1 expression in the mice hearts 8 weeks after MI. However, for the acute MI model, significant increased NEU1 expression was observed in mice in acutely infarct area, but restore baseline 8 weeks after MI. Western blot, RT-PCR, and ELISA data showed the expression of NEU1 were increased in the non-infarct area of mouse hearts 8 weeks after MI (Figures 1D–F). In summary, these data suggested that NEU1 contributed to cardiac ischemic injury at the early stage, as well as chronic pathological after MI. Highly expressing NEU1 accumulate in the non-infarct heart during the chronic phase of MI, suggesting a potential role for NEU1 in regulating cardiac remodeling in an MI setting.

### NEU1 Knockdown Improved Survival Rate and Cardiac Function in Mice Post-MI

**Figure 2:**
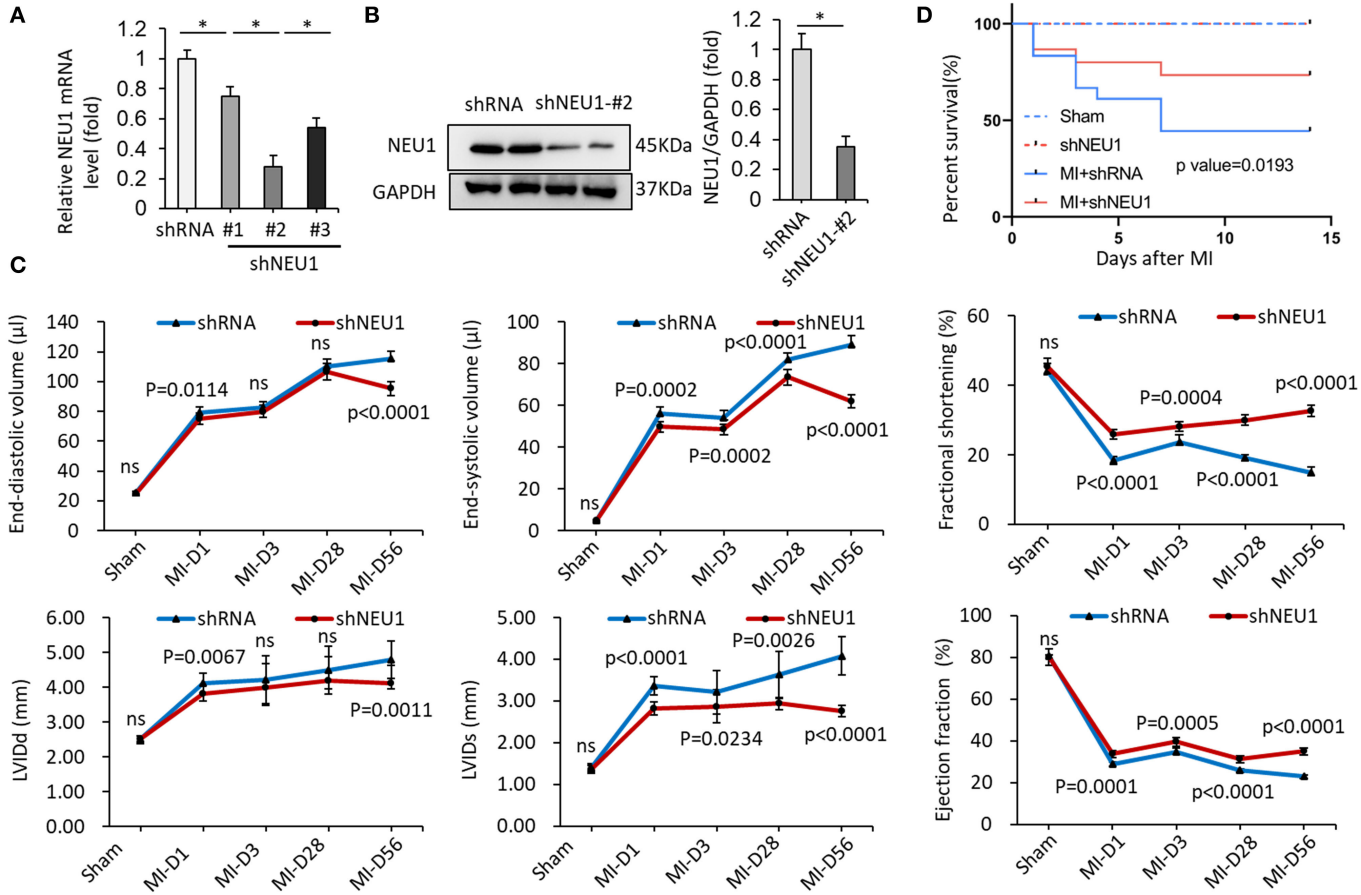
NEU1 knockdown improved survival rates and cardiac function post-MI. **(A)** mRNA levels of NEU1 after AAV9-shRNA or AAV9-shNEU1 injection in mice heart (*n* = 6). **(B)** NEU1 protein expression and quantitative data (*n* = 6). **(C)** Echocardiographic measurements of left ventricular internal diameter at end-systole (LVIDs), left ventricular internal diameter at end-diastole (LVIDd), fractional shortening (FS), ejection fraction (EF), end-diastolic volume (EDV), and end-systolic volume (ESV) in shRNA and shNEU1 hearts after sham operation or 1, 3, 28 and 56 (*n* = 12 per group) days post-MI. **(D)** Kaplan–Meier survival analysis of mice after MI (*n* = 12). *p < 0.05 vs. corresponding group. n.s., non-significant.

Next, we hypothesized that the knockdown of NEU1 may exert functional effects on the chronic phase of post-MI. We used small hairpin RNA (shRNA) delivered by cardiotropic adeno-associated virus (AAV) 9 vectors to cardiac region-specific NEU1 knockdown mice. As shown in Figure 2A, AAV9-shNEU1#2 significantly inhibited NEU1 mRNA level in mice hearts compared with others. The efficiency of AAV9-shNEU1 is presented in Figure 2B. The specific inhibitory expression of NEU1 is achieved in the heart, not in other tissues (lung, liver, etc.) (Supplementary Figure 1A). To determine the functional role of NEU1 in MI tissue, mice were subjected to permanent LAD ligation for 4 weeks after AAV9 injection. At 0, 3, 28, and 56 days after MI, the ejection fraction (EF), fractional shortening (FS), end-systolic volume (ESV), and left ventricular diameter (LVIDs) of shNEU1 mice were significantly better than those of shRNA mice. Besides, LV chamber dilation was attenuated in shNEU1 mic 8 weeks post-MI, as demonstrated by a drastic decline in end diastolic volume (EDV), ESV, LV internal diameter at end diastole (LVIDd), and LVIDs (Supplementary Figure 1B, Figure 2C). Remarkably, knockdown of NEU1 in cardiac robustly improved post-MI survival (Figure 2D). Collectively, our data illustrate the beneficial effects of cardiac-specific NEU1 loss on MI.

### NEU1 Knockdown Prevented Cardiac Hypertrophy and Fibrosis Post-MI

**Figure 3:**
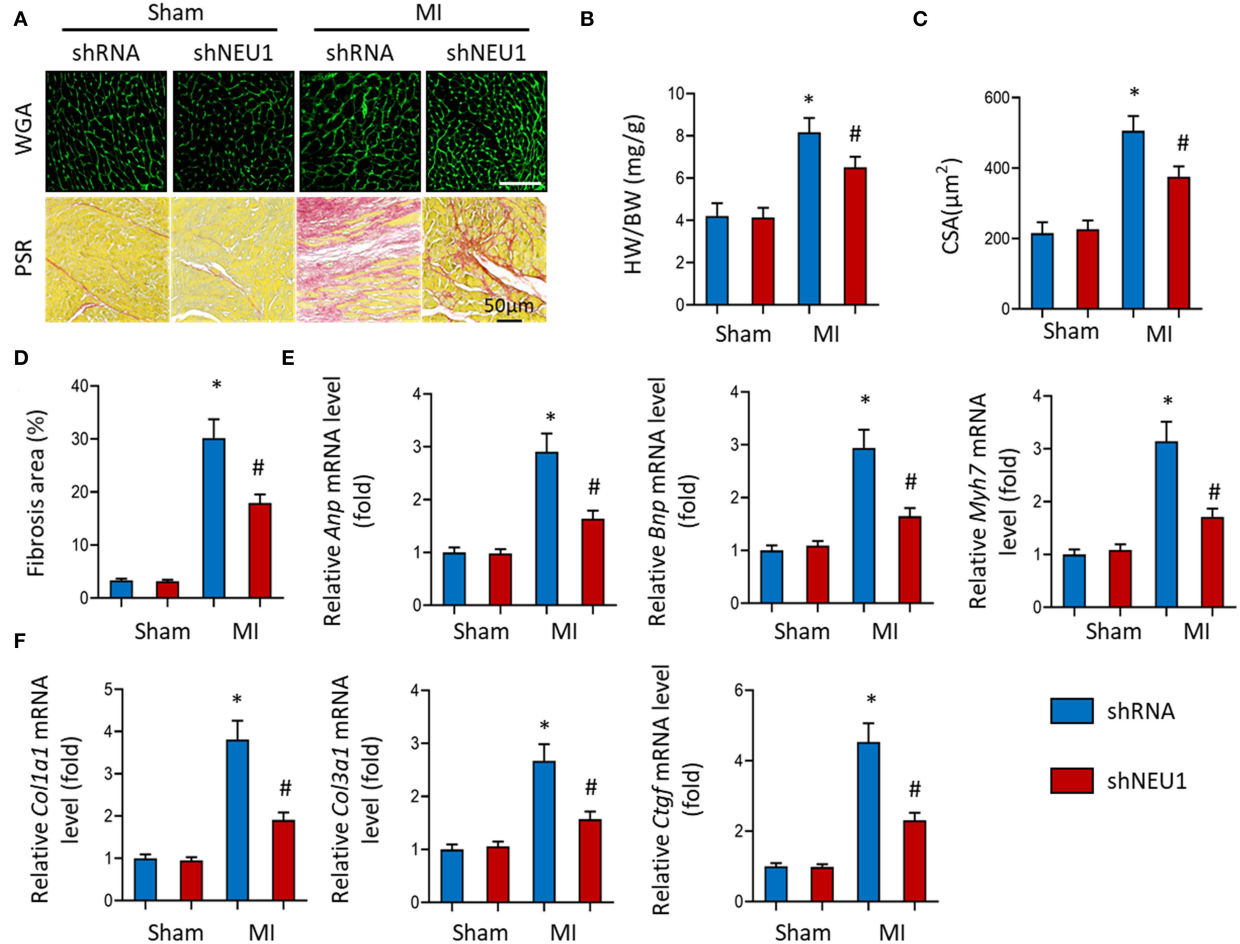
NEU1 knockdown prevented cardiac hypertrophy and fibrosis post-MI. **(A)** WGA staining and PSR staining of shNEU1 and shRNA in mice hearts 8 weeks post MI or sham (*n* = 12). **(B)** Statistical results of heart weight/body weight (HW/BW, *n* = 12). **(C)** Statistical results for the cross-sectional areas of myocytes (CSA, *n* =100 cells/sample, *n* = 6 per group). **(D)** Quantification of fibrotic areas in mice hearts 8 weeks post-MI. **(E,F)** RT-PCR analyses of fetal gene (*Anp, Bnp, Myh7*) and fibrotic markers (*Col1a1, Col3a1, Ctgf*) in each group (*n* = 6). *p < 0.05 vs. corresponding group. n.s., non-significant.

After MI, the infarcted myocardium increases the workload of the non-infarcted myocardium, leading to hypertrophy and fibrosis (35). WGA stained heart sections showed that NEU1 deficiency reduced the CSA of cardiomyocytes (Figures 3A,C). and significantly reduced the heart weight/body weight ratio (HW/BW) (Figure 3B) post-MI. PSR data showed that NEU1 deficiency mice significantly attenuated myocardial fibrosis caused by MI compared with mice injected with shRNA (Figures 3A,D). Compared with the MI+shRNA group, the hypertrophy genes (ANP, BNP, etc.) RNA level was significantly downregulated in the hearts of MI+shNEU1 group mice (Figure 3E). In addition, RT-PCR showed that NEU1 knockdown inhibited MI-induced increases in *Col1a1, Col3a1*, and *Ctgf* (Figure 3F). Collectively, the improvement in heart function in the MI + shNEU1 group of mice was consistent with alleviated cardiac remodeling as evidenced by suppression of cardiac hypertrophy and fibrosis in infarcted hearts.

### NEU1 Knockdown Attenuated Mitochondrial Deficiencies and Enhanced SIRT1/PGC-1α Pathway Activation Post-MI

**Figure 4:**
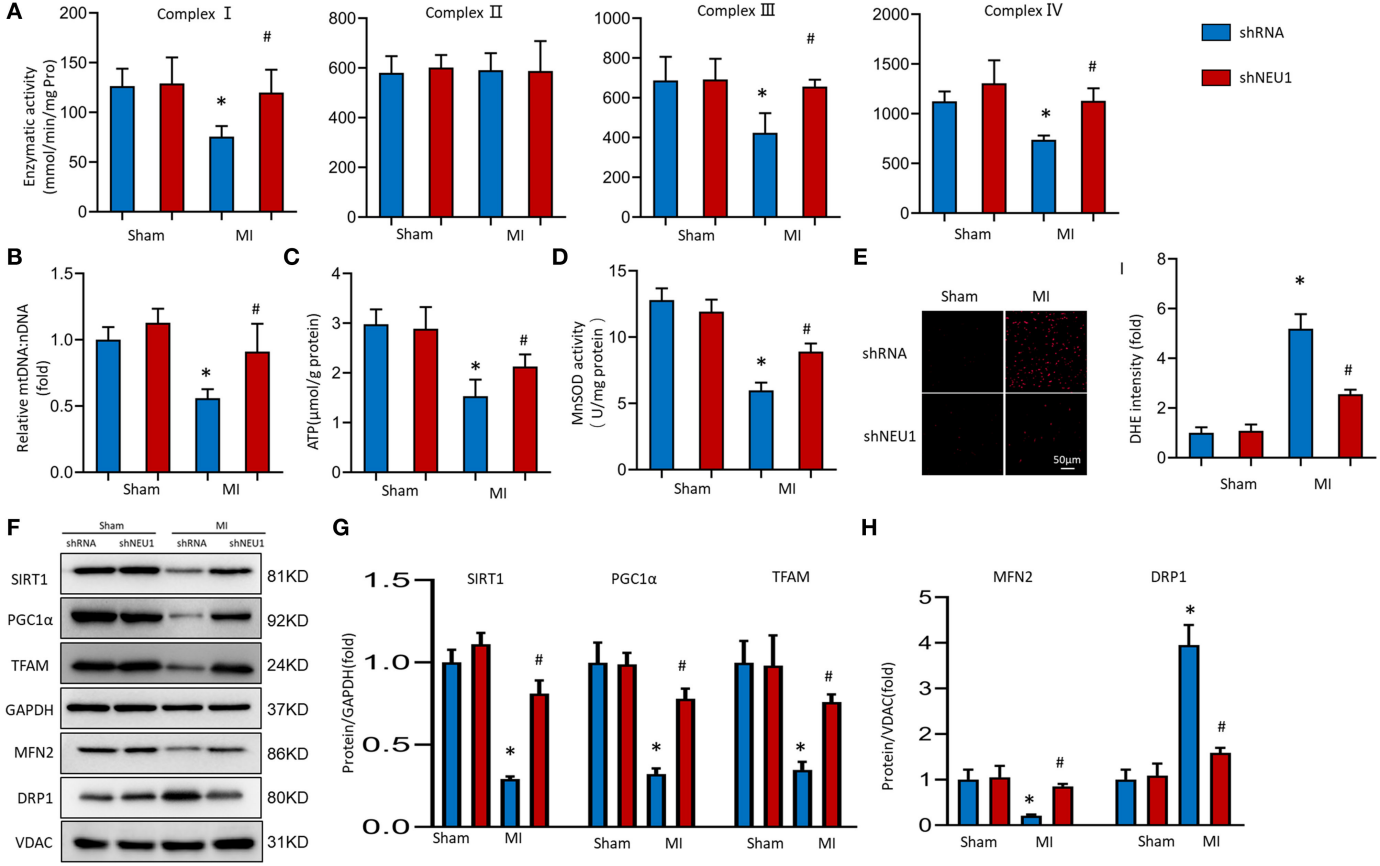
NEU1 knockdown attenuated mitochondrial deficiencies and ROS accumulation post-MI. **(A)** Enzymatic activity of mitochondrial electron transport chain enzymes (I, II, III, and IV) in isolated mitochondria from four groups of mice (*n* = 6). **(B)** Relative mitochondrial DNA content (mtDNA: nDNA) of heart (*n* = 6). **(C)** ATP content of cardiomyocytes in fresh heart (*n* = 6). **(D)** Mitochondrial ROS is assessed by MnSOD activity of fresh heart (*n* = 6). **(E)** ROS production in myocardial detected by DHE in fresh heart tissues. **(F–H)** Western blot image and quantitative results of the SIRT1/PGC1α pathway, the MFN2, and DRP1 of each group *in vivo* (*n* = 6). *p < 0.05 vs. Sham+shRNA group, #*p* < 0.05 vs. MI+shRNA group.

Mitochondrial dysfunction and structural changes that occur during ischemia are critically implicated in pathophysiology in the infarcted myocardium (36). We measured the respiratory chain enzyme activities. Under basic conditions, the mitochondrial respiratory chain complexes enzyme activities in the Sham group and shNEU1 group did not change significantly, while the enzyme activities of complexes I, III, and IV in the MI group were significantly reduced when compared with the Sham group. Most importantly, the enzyme activity of complex II did not decrease significantly in the shNEU1-MI group because it is only encoded by nuclear DNA (Figure 4A). mtDNA copy number data showed that, the mtDNA:nDNA ratio in the MI group was reduced when compared with the Sham group, while the NEU1 knockdown increased the ratio (Figure 4B). ATP, as the most important energy molecule, plays an important role in various physiological and pathological processes of cardiomyocytes. The significant decrease in ATP levels of ischemic myocardium indicates impaired or decreased mitochondrial function, and is related to the irreversible changes in myocardial cells because the cells exhaust their energy storage and cannot sustain cellular vital activities (35). ATP content was significantly lower in 8 weeks post-MI mice hearts, and NEU1 knockdown significantly increased the ATP content in the context of MI (Figure 4C). Manganese superoxide dismutase (SOD2 or MnSOD) is a mitochondrial matrix antioxidant enzyme responsible for removing free radicals generated locally (37). The activity of MnSOD decreased in 8 weeks post-MI, while NUE1 inhibition can improve the decreased MnSOD activity induced by MI (Figure 4D). Mitochondria are responsible for the production of ATP, which can produce a small amount of ROS. Therefore, we determined the mitochondrial ROS production in mice hearts. NEU1 knockdown attenuated oxidative stress in mice hearts with MI surgery (Figure 4E).

Transcription factor A plays a vital role in the maintenance of mtDNA and thus, ATP production (38). SIRT1 and PGC-1α are key regulators involved in processes such as myocardial mitochondrial biogenesis and energy metabolism. Western blots showed that SIRT1, PGC-1α, and TFAM expression levels decreased after MI surgery for 8 weeks. Meanwhile, NEU1 knockdown increased cardiac expression of these proteins in MI mice (Figures 4F,G). The imbalance between mitochondrial division and fusion is closely related to the pathology of myocardial I/R injury (39). MFN2 and DRP1 are essential mitochondrial protein, which mediates mitochondrial functions. The expression of MFN2 decreased, and the expression of DRP1 increased in MI tissues, however, NEU1 knockdown reversed the above-mentioned protein changes (Figures 4F,H). In summary, these findings indicate that NEU1 inhibition alleviated ischemia-associated mitochondrial damage and exerted cardioprotective effects by enhancing the SIRT1/PGC-1α signaling pathway *in vivo*.

### NEU1 Inhibition Attenuated Hopoxia-Induced Mitochondrial Deficiencies and Enhanced SIRT1/PGC-1α Pathway Activation *in vitro*

**Figure 5:**
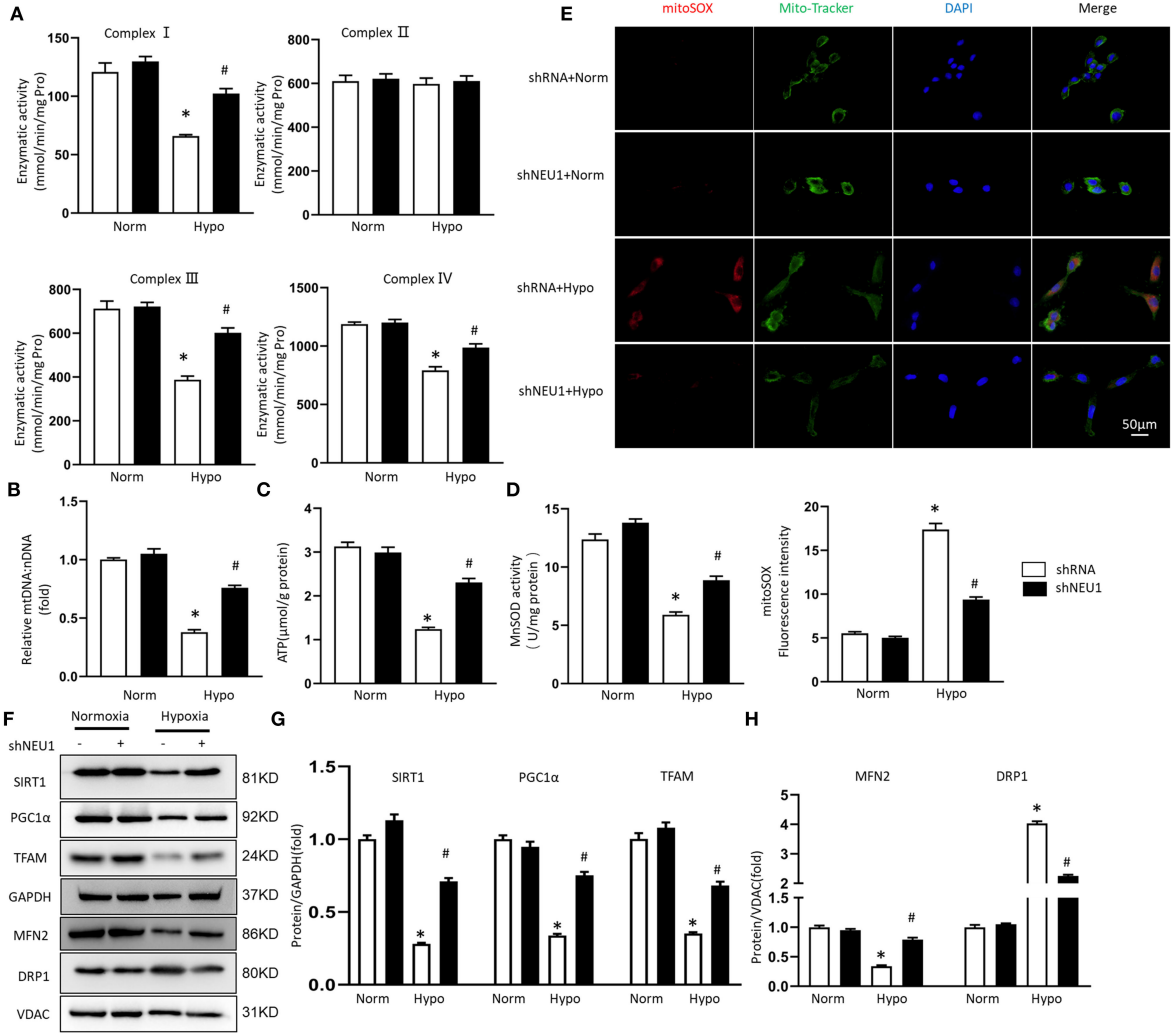
NEU1 knockdown attenuated mitochondrial deficiencies and ROS accumulation post-hypoxia *in vitro*. **(A)** Enzymatic activity of mitochondrial electron transport chain enzymes (I, II, III, and IV) in isolated mitochondria from four groups of NRCMs (*n* = 6). **(B)** Relative mitochondrial DNA content (mtDNA: nDNA) of NRCMs (*n* = 6). **(C)** ATP content of NRCMs in each group (*n* = 6). **(D)** Mitochondrial ROS is assessed by MnSOD activity of NRCMs (*n* = 6). **(E)** Superoxide production in mitochondria detected by MitoSOX staining in NRCMs. **(F–H)** Western blot image and quantitative results of the SIRT1/PGC1α pathway, the MFN2 and DRP1 of each group in H9C2 cells (*n* = 6). *p < 0.05 vs. normoxia + shRNA group, #*p* < 0.05 vs. hypoxia + shRNA group.

We transfected NRCMs with shRNA or shNEU1 and then cultured NRCMs in a hypoxia or normoxia environment. There are no significant changes in the enzymatic activity of complex I, II, III, and IV between normxia+shRNA group and normxia+shNEU1 group. Hypoxia treatment significantly decreased the enzymatic activities of complex I, III, and IV, while NEU1 inhibition blocked these enzyme activity changes. Consistent with results *in vivo*, the enzymatic activity of complex II was not altered in either group (Figure 5A). mtDNA copy number data showed that compared with the normxia+shRNA group, the mtDNA:nDNA ratio in the hypoxic+shRNA group decreased, while NEU1 knockdown increased the ratio (Figure 5B). ATP content and MnSOD activity were significantly decreased by administrated with hypoxia but increased with NEU1 inhibition (Figures 5C,D). The fluorescence intensity of the mitoSOX among groups proved that NEU1 knockdown attenuated oxidative stress in NRCMs with hypoxia treatment (Figure 5E).

Consistent with *in vivo* experiments, hypoxia treatment significantly decreased SIRT1, PGC-1α, and TFAM protein levels, but increased by adding of shNEU1 in H9C2 cells (Figures 5F,G). Hypoxia decreased the MFN2 level and increased the DRP1 level, however, NEU1 knockdown increased the MFN2 level and decreased the DRP1 level in H9C2 cells (Figures 5F,H).

### SIRT1 or PGC-1α Inhibition Abolished ShNEU1-Mediated Mitochondrial Biogenesis and Function Improvement in Hypoxia-Administrated NRCMs or H9C2 Cells

**Figure 6:**
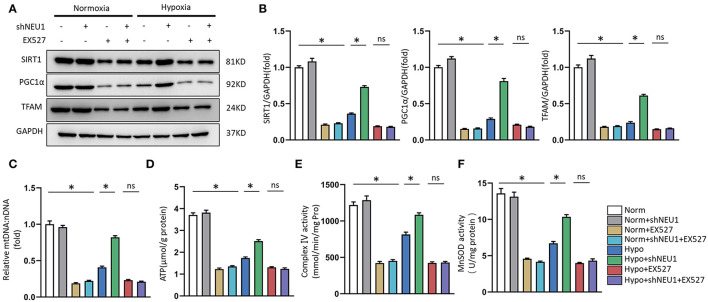
SIRT1 inhibition abolished shNEU1-induced mitochondrial biogenesis and function improvement in hypoxia-administrated NRCMs or H9C2 cells. **(A,B)** Western blot image and quantitative results of SIRT1, PGC1α and TFAM of each group in H9C2 cells (*n* = 6). **(C)** Relative mitochondrial DNA content (mtDNA: nDNA) of NRCMs (*n* = 6). **(D)** ATP content of NRCMs (*n* = 6). **(E)** Enzymatic activity of mitochondrial complex IV in isolated mitochondria from four groups of NRCMs (*n* = 6). **(F)** Mitochondrial ROS is assessed by MnSOD activity of NRCMs (*n* = 6). *p < 0.05 vs. corresponding group, n.s., non-significant.

Use SIRT1 inhibitor EX-527 to verify whether shNEU1-mediated mitochondrial metabolism and functional enhancement are mediated by SIRT1 activation. As shown in Figures 6A,B, shNEU1 enhanced the SIRT1, PGC-1α, and TFAM protein levels in H9C2 cells cultured under a hypoxia environment. Meanwhile, EX527 eliminated the increase in these protein expressions induced by shNEU1. We next detected the mtDNA level, the content of ATP production, complex IV activity, and mitochondrial oxidative stress in NRCMs. We noted that NEU1 inhibition reversed the hypoxia-induced decrease in mtDNA level, ATP levels, complex IV activity, and MnSOD activity, whereas EX527 partly abrogated these effects (Figures 6C–F).

**Figure 7:**
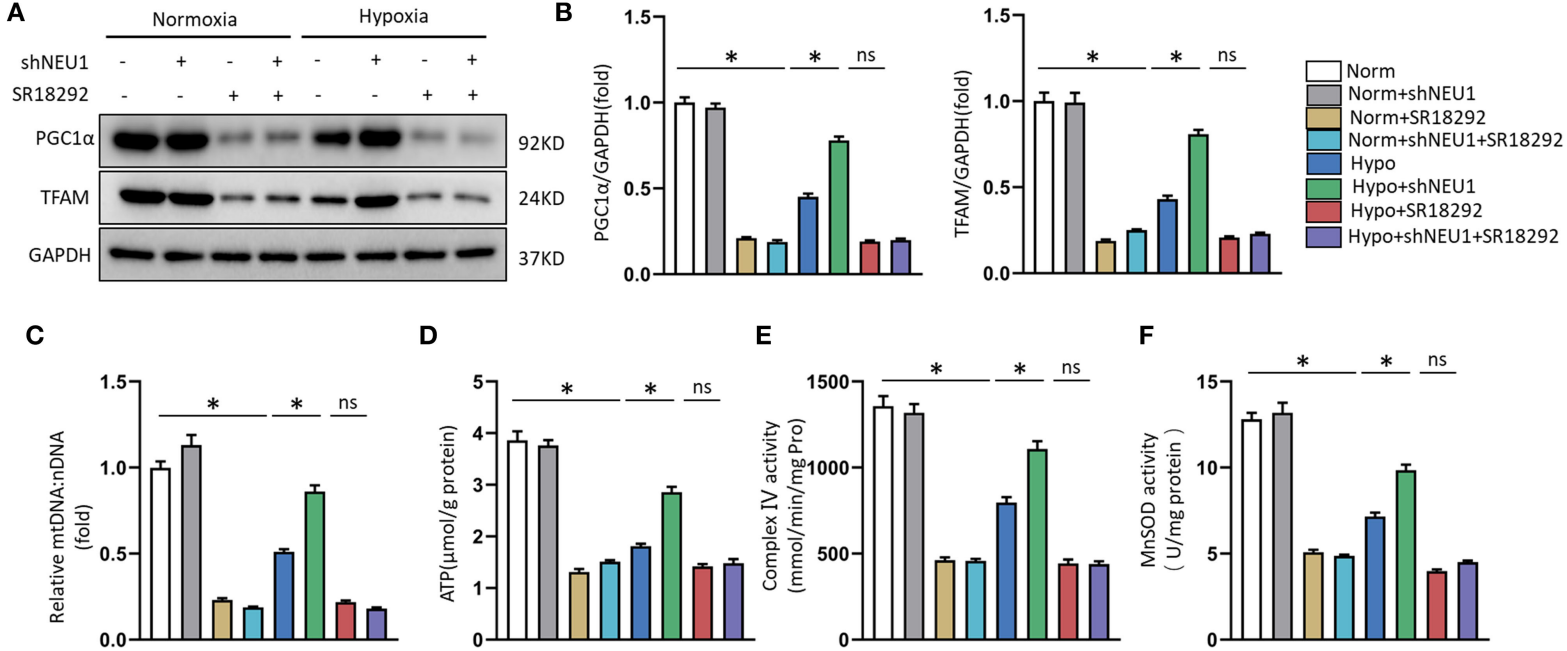
PGC1α inhibition abolished shNEU1-induced mitochondrial biogenesis and function improvement in hypoxia-administrated NRCMs or H9C2 cells. **(A,B)** Western blot image and quantitative results of PGC1α and TFAM of each group in H9C2 cells (*n* = 6). **(C)** Relative mitochondrial DNA content (mtDNA: nDNA) of NRCMs (*n* = 6). **(D)** ATP content of NRCMs (*n* = 6). **(E)** Enzymatic activity of mitochondrial complex IV in isolated mitochondria from four groups of NRCMs (*n* = 6). **(F)** Mitochondrial ROS is assessed by MnSOD activity of NRCMs (*n* = 6). *p < 0.05 vs. corresponding group, n.s., non-significant.

Next, to test whether that PGC-1α is a key regulator in shNEU1-mediated mitochondrial biogenesis and functional improvement, we examined the alterations in PGC-1α and TFAM through exposure to the PGC-1α inhibitor SR-18292. Similar to EX-527, the PGC-1α inhibitor abolished the shNEU1-induced increase in protein expressions in H9C2 cells (Figures 7A,B). We next detected the expression of mtDNA, the content of ATP production, complex IV activity, and mitochondrial oxidative stress in NRCMs. We noted that NEU1 inhibition reversed the hypoxia-induced decrease in mtDNA level, ATP levels, complex IV activity, and MnSOD activity, whereas SR-18292 partly abrogated these effects (Figures 7C–F). These results strongly suggest that shNEU1 relieves MI by activating SIRT1/PGC-1α and enhancing mitochondrial biogenesis, thereby improving cardiomyocyte mitochondrial function.

### PGC1α Deficiency Offset the Cardio-Protective Effects of NEU1 Knockdown *in vivo*

**Figure 8:**
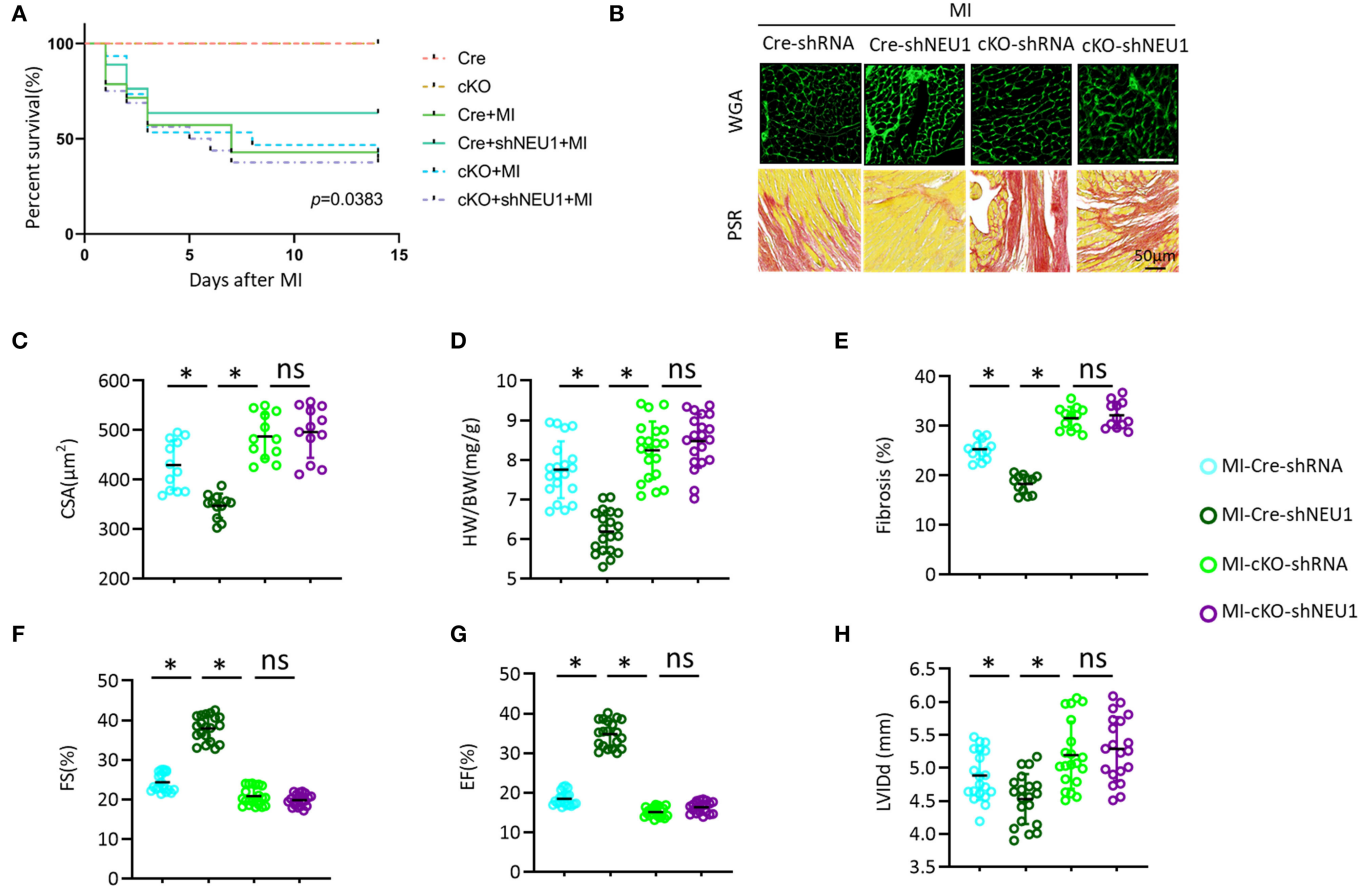
PGC1α deficiency offset the protective effects of shNEU1 *in vivo*. **(A)** Kaplan–Meier survival analysis of each group in 2 weeks after MI (*n* = 20). **(B)** WGA staining and PSR staining of each group mice heart (*n* = 12). **(C)** Statistical results for the cross-sectional areas of myocytes (CSA, *n* =100 cells/sample, *n* = 12 per group). **(D)** Statistical results of HW/BW (*n* = 20). **(E)** Quantification of fibrotic areas in 8 weeks post-MI (*n* = 12). **(F)** FS of mice in 8 weeks post MI (*n* = 20). **(G)** EF of mice in 8 weeks after MI (*n* = 20). **(H)** LVIDd diameter (*n* = 20). *P < 0.05 vs. corresponding group; n.s., non-significant.

To explore its mechanism more deeply, we further used PGC-1α-cKO mice to study the effects of PGC-1α on shNEU1-induced cardioprotection. Consistent with *in vitro* experimental data, we observed that NEU1 inhibition lost the protective effect in PGC-1α-cKO mice, as evidenced by the indistinguishable survival rate between the cKO+MI group and cKO+MI+shNEU1 group (Figure 8A). Besides, WGA staining and HW/BW results showed that NEU1 knockdown does not counter myocardial hypertrophy induced by MI in PGC-1α-cKO mice (Figures 8B–D). PSR staining results showed that NEU1 inhibition cannot alleviate cardiac fibrosis in consistent with the findings *in vitro*, shNEU1 lost its protective effects on inflammatory response in cKO+MI+shNEU1 group mice (Figures 8B,E). Furthermore, the heart function (determined by FS, EF, and LVIDd), showed no significant differences between the cKO+MI group and the cKO+MI+shNEU1 group (Figures 8F–H). Collectively, these data indicated that PGC-1α ablation completely eliminated the cardioprotection of NEU1 knockdown.

## Discussion

In this study, we demonstrated a novel role for NEU1 in MI. We observed that NEU1 is significantly elevated in mice hearts post-MI. Cardiac region-specific NEU1 inhibition prevented the development of cardiac dysfunction and remodeling in chronic MI hearts, *via* improving mitochondrial energy metabolism and decreasing mitochondrial oxidative stress in myocardial tissue post-MI. Mechanistically, through *in vivo* and *in vitro* experiments, NEU1 knockdown ameliorated cardiomyocytes injury by regulating the SIRT1/PGC-1α signaling pathway, thereby enhancing the biogenesis and function of mitochondria. Together, these data reveal a previously unappreciated mechanism that governs mitochondrial energy metabolism switch and chronic MI-induced cardiac remodeling.

In our study, we found that NEU1 is dramatically elevated in the infarct area of acute ischemic cardiac injury, which is consistent with previous findings (34). However, the increased NEU1 in the infarct area was declined to the baseline during the chronic phase of MI. In the infarcted area, scar tissue was formed during chronic MI, with few viable cardiomyocytes, leading to NEU1 decreasing. While, for the chronic MI model, NEU1 expression was significantly increased in non-infarct areas of mice, suggesting a potential role of NEU1 in regulating MI-induced cardiac remodeling.

As a member of the neuraminidases family, NEU1 is known to participate in multiple cellular processes (26, 40–42) and inherited disease (43). Collectively, NEU1 is closely related to inflammation and promotes the occurrence and development of atherosclerosis and HF (44). In the human hypertrophic cardiomyopathy or rodent myocardial hypertrophy model, NEU1 was significantly increased, and targeted inhibition of NEU1 expression effectively prevented the development of cardiac hypertrophy and remodeling (22). There was a similar article recently published mentioned that after the myocardium is exposed to I/R, the NEU1 protein level and activity in myocardial cells and infiltrating monocytes increase, and cause inflammation, hypertrophy, and HF. However, systemic inhibition of NEU1 can alleviates myocardial injury and dysfunction after I/R injury (20). Similar to this study, the expression of NEU1 is elevated in myocardial tissue of MI, and NEU1 knockdown can significantly improve cardiac dysfunction and myocardial remodeling. Importantly, the difference and novelty of our work are that, NEU1 knockdown is a protective effect produced by improving cardiac mitochondrial dysfunction and mitochondrial oxidative stress. While the previous research direction is that NEU1 promotes the increased level and duration of monocyte inflammation after reperfusion of an infarcted heart. In our hands, NEU1 level was increased in the ischemic heart while NEU1 inhibition preserved cardiac function and improved myocardial morphology post-MI.

More and more evidence showed that SIRT1 can induce nuclear localization and deacetylation to increase the transcriptional activity of PGC-1α, and has an important role in promoting the function and structure of mitochondria (45, 46), which is associated with improved metabolic regulation and antioxidant stress (47). PGC-1α is a key regulator of mitochondrial structure and function, which controls the expression of mitochondrial and nuclear-encoding mitochondrial genes, and regulates the transcription of TFAM (48). In fact, overexpression of SIRT1 and subsequent activation of PGC-1α protects against metabolic decline and cardiovascular disease (49, 50). In our work, we found that under MI or hypoxia conditions, the SIRT1, PGC-1α, and TFAM levels downregulated significantly. While NEU1 knockdown prevented the decline of SIRT1/PGC-1α signaling proteins in MI, or in hypoxia-treated H9C2 cells. Since the inhibition of SIRT1/PGC-1α axis protein by NEU1 was eliminated by independent inhibition of SIRT1 activity (EX527) or PGC-1α expression (SR-18292). Our data show that there is a strong correlation between NEU1 and the mitochondrial biogenesis mediated by the SIRT1/PGC-1α pathway, and it has an important potential impact on myocardial remodeling during MI. Since the effects of NEU1 inhibition on SIRT1/PGC-1α axis protein are eliminated by independently inhibiting SIRT1 activity (EX527) or PGC-1α expression (with SR-18292), our data indicated that there is a strong association between SIRT1/PGC-1α pathway-mediated NEU1 and mitochondrial biogenesis, with important potential implications for HF post-MI.

A large number of reports have revealed the adverse effects of mitochondrial damage in the onset and progression of acute MI (51), which is well-related to our results. Previous studies have shown a close link between mtDNA damage and reduced mitochondrial electron transport complex enzyme activity (52). Because the maintenance of mtDNA is essential for mitochondrial protein expression, the reduction of mtDNA copy number can lead to mitochondrial dysfunction and loss. Evidence shows that mtDNA defects, mitochondrial structural changes and dysfunction promote the occurrence and progression of HF (53). PGC-1α acts on the upstream of TFAM and can increase mtDNA levels in cells and mice (54). Our study showed that the relative amounts of TFAM and mtDNA decreased significantly in the mouse heart failure model after MI, and clearly proved that NEU1 inhibition can limit the decline of mtDNA levels and keep it at a normal level in the heart tissue of MI mice. Consistent with the changes in mtDNA and transcription levels, the activity of complexes I, III, and IV is significantly reduced in the heart after MI because part of it is encoded by the mtDNA gene; while the complex II activity is not affected because it is entirely composed of nuclear DNA coding. Knockdown of NEU1 can significantly improve the adverse changes in the mitochondrial complexes I, III, and IV activity caused by MI or hypoxia. Transgene expression of PGC-1α or the use of peroxisome proliferator-activated receptor agonist benzoate to treat mitochondrial myopathy can promote mitochondrial biogenesis, improve respiration, and prolong lifespan (55). We found that inhibition of NEU1 maintained the ATP levels of cardiomyocytes in MI, and these beneficial outcomes were eliminated independently by EX527 or SR-18292.

The stability of the dynamic balance between mitochondrial fusion and division is very important to maintain the biological function of mitochondria (56). MFN2 and DRP1 are important proteins regulating mitochondrial fusion and division (56, 57), and MFN2 can participate in cell proliferation and apoptosis and maintains mitochondrial DNA stability by regulating mitochondrial fusion and division and changing the morphology and function of mitochondria (58). Overexpression of DRP1 protein can promote mitochondrial division and damage the mitochondrial network structure. Inhibition of DRP1 promotes mitochondrial fusion, increases network structure, and repairs damaged mitochondria (59). In this study, the MFN2 protein level was decreased and the DRP1 protein level was increased in the pathological state, suggesting that MI-induced decreased release of the mitochondrial fusion protein and increased production of mitotic protein. While NEU1 knockdown blocks undesirable changes in mitochondrial fusion and division proteins, suggesting that NEU1 inhibition may prevent mitochondrial damage by promoting MFN2 expression and inhibiting DRP1 expression, inhibiting the occurrence of mitochondrial division.

This study has some limitations. First, NEU1 was elevated in MI mice hearts, while, we did not explore why and how NEU1 increased. Second, in our work, we mainly used AAV9-NEU1 to verify its effect, and did not use genetically engineered mice related to NEU1. Third, the study only investigated the inhibitory effect of NEU1 but did not study the role of overexpression of NEU1.

In summary, the evidence we provide shows that the expression of NEU1 in the myocardial tissue of MI mice is significantly upregulated, and the biogenesis and function of mitochondria are impaired. At the same time, the signal molecules in the SIRT1/PGC-1α pathway are downregulated. Through *in vivo* and *in vitro* experimental analysis, we proved that NEU1 inhibition promotes the biogenesis and function of mitochondria by enhancing the SIRT1/PGC-1α signaling pathway, thereby improving poor myocardial remodeling after MI. NEU1 inhibition opens up a whole new field of treatment after myocardial infarction. Existing NEU1 inhibitors (antiviral drugs such as zanamivir and oseltamivir) have good safety and pharmacokinetic properties and may be used to ameliorate mitochondrial metabolism and oxidative stress in myocardial infarction or heart failure.

## Data Availability Statement

The original contributions presented in the study are included in the article/Supplementary Materials, further inquiries can be directed to the corresponding author/s.

## Ethics Statement

The animal study was reviewed and approved by the Animal Care and Use Committee of Renmin Hospital of Wuhan University (IACUC Issue No. WDRM20190803).

## Author Contributions

ZG, F-YL, and DF performed experiments and analyzed the data. ZG, DF, ZY, and Q-ZT designed experiments, supervised and conceptualized the study, and wrote and edited the manuscript. ZG and DF wrote and edited the manuscript. S-QM, PA, DY, and M-YW assisted with some experiments and discussed the results. ZG and F-YL conducted bioinformatics analysis. All authors contributed to the article and approved the submitted version.

## Funding

The National Natural Science Foundation of China (81900219).

## Conflict of Interest

The authors declare that the research was conducted in the absence of any commercial or financial relationships that could be construed as a potential conflict of interest.

## Publisher's Note

All claims expressed in this article are solely those of the authors and do not necessarily represent those of their affiliated organizations, or those of the publisher, the editors and the reviewers. Any product that may be evaluated in this article, or claim that may be made by its manufacturer, is not guaranteed or endorsed by the publisher.

## References

[1] Benjamin EJ Virani SS Callaway CW Chamberlain AM Chang AR Cheng S. Heart disease and stroke statistics-2018 update: a report from the American Heart Association. Circulation. (2018) 137:e67–e492. 10.1161/CIR.0000000000000573 29386200

[2] Owens AT Brozena SC Jessup M. New management strategies in heart failure. Circ Res. (2016) 118:480–95. 10.1161/CIRCRESAHA.115.306567 26846642

[3] Braunwald E. The war against heart failure: the Lancet lecture. Lancet. (2015) 385:812–24. 10.1016/S0140-6736(14)61889-4 25467564

[4] Cohn WE Timms DL Frazier OH. Total artificial hearts: past, present, and future. Nat Rev Cardiol. (2015) 12:609–17. 10.1038/nrcardio.2015.79 26031698

[5] Yacoub M. Cardiac donation after circulatory death: a time to reflect. Lancet. (2015) 385:2554–6. 10.1016/S0140-6736(15)60683-3 25888087

[6] Del Re DP Amgalan D Linkermann A Liu Q Kitsis RN. Fundamental mechanisms of regulated cell death and implications for heart disease. Physiol Rev. (2019) 99:1765–817. 10.1152/physrev.00022.2018 31364924 PMC6890986

[7] Davidson SM Adameova A Barile L Cabrera-Fuentes HA Lazou A Pagliaro P. Mitochondrial and mitochondrial-independent pathways of myocardial cell death during ischaemia and reperfusion injury. J Cell Mol Med. (2020) 24:3795–806. 10.1111/jcmm.15127 32155321 PMC7171390

[8] Gibb AA Hill BG. Metabolic coordination of physiological and pathological cardiac remodeling. Circ Res. (2018) 123:107–28. 10.1161/CIRCRESAHA.118.312017 29929976 PMC6023588

[9] Siasos G Tsigkou V Kosmopoulos M Theodosiadis D Simantiris S Tagkou NM. Mitochondria and cardiovascular diseases-from pathophysiology to treatment. Ann Transl Med. (2018) 6:256. 10.21037/atm.2018.06.21 30069458 PMC6046286

[10] Eltzschig HK Eckle T. Ischemia and reperfusion–from mechanism to translation. Nat Med. (2011) 17:1391–401. 10.1038/nm.2507 22064429 PMC3886192

[11] Newmeyer DD Ferguson-Miller S. Mitochondria: releasing power for life and unleashing the machineries of death. Cell. (2003) 112:481–90. 10.1016/S0092-8674(03)00116-8 12600312

[12] Choi HI Kim HJ Park JS Kim IJ Bae EH Ma SK. PGC-1alpha attenuates hydrogen peroxide-induced apoptotic cell death by upregulating Nrf-2 *via* GSK3beta inactivation mediated by activated p38 in HK-2 Cells. Sci Rep. (2017) 7:4319. 10.1038/s41598-017-04593-w 28659586 PMC5489530

[13] McDermott-Roe C Ye J Ahmed R Sun XM Serafin A Ware J. Endonuclease G is a novel determinant of cardiac hypertrophy and mitochondrial function. Nature. (2011) 478:114–8. 10.1038/nature10490 21979051 PMC3189541

[14] Vina J Gomez-Cabrera MC Borras C Froio T Sanchis-Gomar F Martinez-Bello VE. Mitochondrial biogenesis in exercise and in ageing. Adv Drug Deliv Rev. (2009) 61:1369–74. 10.1016/j.addr.2009.06.006 19716394

[15] Gomes AP Price NL Ling AJ Moslehi JJ Montgomery MK Rajman L. Declining NAD(+) induces a pseudohypoxic state disrupting nuclear-mitochondrial communication during aging. Cell. (2013) 155:1624–38. 10.1016/j.cell.2013.11.037 24360282 PMC4076149

[16] Gerhart-Hines Z Rodgers JT Bare O Lerin C Kim SH Mostoslavsky R. Metabolic control of muscle mitochondrial function and fatty acid oxidation through SIRT1/PGC-1alpha. EMBO J. (2007) 26:1913–23. 10.1038/sj.emboj.7601633 17347648 PMC1847661

[17] Yuan Y Cruzat VF Newsholme P Cheng J Chen Y Lu Y, Regulation Regulation of SIRT1 in aging: Roles in mitochondrial function and biogenesis. Mech Ageing Dev. (2016) 155:10–21. 10.1016/j.mad.2016.02.003 26923269

[18] Miyagi T Yamaguchi K. Mammalian sialidases: physiological and pathological roles in cellular functions. Glycobiology. (2012) 22:880–96. 10.1093/glycob/cws057 22377912

[19] Quach ME Chen W Li R. Mechanisms of platelet clearance and translation to improve platelet storage. Blood. (2018) 131:1512–21. 10.1182/blood-2017-08-743229 29475962 PMC5887765

[20] Heimerl M Sieve I Ricke-Hoch M Erschow S Battmer K Scherr M. Neuraminidase-1 promotes heart failure after ischemia/reperfusion injury by affecting cardiomyocytes and invading monocytes/macrophages. Basic Res Cardiol. (2020) 115:62. 10.1007/s00395-020-00821-z 32975669 PMC7519006

[21] Hanson VA Shettigar UR Loungani RR Nadijcka MD. Plasma sialidase activity in acute myocardial infarction. Am Heart J. (1987) 114:59–63. 10.1016/0002-8703(87)90307-3 3604873

[22] Chen QQ Ma G Liu JF Cai YY Zhang JY Wei TT. Neuraminidase 1 is a driver of experimental cardiac hypertrophy. Eur Heart J. (2021) 42:3770–82. 10.1093/eurheartj/ehab347 34179969

[23] Gao E Lei YH Shang X Huang ZM Zuo L Boucher M. A novel and efficient model of coronary artery ligation and myocardial infarction in the mouse. Circ Res. (2010) 107:1445–53. 10.1161/CIRCRESAHA.110.223925 20966393 PMC3005817

[24] Yan W Lin C Guo Y Chen Y Du Y Lau WB. N-Cadherin overexpression mobilizes the protective effects of mesenchymal stromal cells against ischemic heart injury through a beta-catenin-dependent manner. Circ Res. (2020) 126:857–74. 10.1161/CIRCRESAHA.119.315806 32079489

[25] Ma SQ Guo Z Liu FY Hasan SG Yang D Tang N. 6-Gingerol protects against cardiac remodeling by inhibiting the p38 mitogen-activated protein kinase pathway. Acta Pharmacol Sin. (2021) 42:1575–86. 10.1038/s41401-020-00587-z 33462378 PMC8463710

[26] Marcu R Neeley CK Karamanlidis G Hawkins BJ, Multi-parameter Multi-parameter measurement of the permeability transition pore opening in isolated mouse heart mitochondria. J Vis Exp. (2012) 67. 10.3791/4131 22987105 PMC3490266

[27] Kramer KA Oglesbee D Hartman SJ Huey J Anderson B Magera MJ. Automated spectrophotometric analysis of mitochondrial respiratory chain complex enzyme activities in cultured skin fibroblasts. Clin Chem. (2005) 51:2110–6. 10.1373/clinchem.2005.050146 16141288

[28] Miro O Barrientos A Alonso JR Casademont J Jarreta D Urbano-Marquez A. Effects of general anaesthetic procedures on mitochondrial function of human skeletal muscle. Eur J Clin Pharmacol. (1999) 55:35–41. 10.1007/s002280050589 10206082

[29] Krahenbuhl S Talos C Wiesmann U Hoppel CL. Development and evaluation of a spectrophotometric assay for complex III in isolated mitochondria, tissues and fibroblasts from rats and humans. Clin Chim Acta. (1994) 230:177–87. 10.1016/0009-8981(94)90270-4 7834868

[30] Veitch K Hue L. Flunarizine and cinnarizine inhibit mitochondrial complexes I and II: possible implication for parkinsonism. Mol Pharmacol. (1994) 45:158–63. 8302275

[31] Guo Z Tang N Liu FY Yang Z Ma SQ An P. TLR9 deficiency alleviates doxorubicin-induced cardiotoxicity *via* the regulation of autophagy. J Cell Mol Med. (2020) 24:10913–23. 10.1111/jcmm.15719 33140921 PMC7521247

[32] Wang J Li S Wang J Wu F Chen Y Zhang H. Spermidine alleviates cardiac aging by improving mitochondrial biogenesis and function. Aging. (2020) 12:650–71. 10.18632/aging.102647 31907336 PMC6977682

[33] Li Y Feng YF Liu XT Li YC Zhu HM Sun MR, Songorine promotes cardiac mitochondrial biogenesis *via* Nrf2 induction during sepsis. Redox Biol. (2021) 38:101771. 10.1016/j.redox.2020.101771 33189984 PMC7674615

[34] Zhang L Wei TT Li Y Li J Fan Y Huang FQ. Functional metabolomics characterizes a key role for n-acetylneuraminic acid in coronary artery diseases. Circulation. (2018) 137:1374–90. 10.1161/CIRCULATIONAHA.117.031139 29212895

[35] Frangogiannis NG. Pathophysiology of Myocardial Infarction. Compr Physiol. (2015) 5:1841–75. 10.1002/cphy.c150006 26426469

[36] Ong SB Gustafsson AB. New roles for mitochondria in cell death in the reperfused myocardium. Cardiovasc Res. (2012) 94:190–6. 10.1093/cvr/cvr312 22108916 PMC3331612

[37] Fukai T Ushio-Fukai M. Superoxide dismutases: role in redox signaling, vascular function, and diseases. Antioxid Redox Signal. (2011) 15:1583–606. 10.1089/ars.2011.3999 21473702 PMC3151424

[38] Kang D Kim SH Hamasaki N. Mitochondrial transcription factor A (TFAM): roles in maintenance of mtDNA and cellular functions. Mitochondrion. (2007) 7:39–44. 10.1016/j.mito.2006.11.017 17280879

[39] Maneechote C Palee S Chattipakorn SC Chattipakorn N. Roles of mitochondrial dynamics modulators in cardiac ischaemia/reperfusion injury. J Cell Mol Med. (2017) 21:2643–53. 10.1111/jcmm.13330 28941171 PMC5661112

[40] Amith SR Jayanth P Franchuk S Finlay T Seyrantepe V Beyaert R. NEU1 desialylation of sialyl alpha-2,3-linked beta-galactosyl residues of TOLL-like receptor 4 is essential for receptor activation and cellular signaling. Cell Signal. (2010) 22:314–24. 10.1016/j.cellsig.2009.09.038 19796680

[41] Hinek A Bodnaruk TD Bunda S Wang Y Liu K. Neuraminidase-1, a subunit of the cell surface elastin receptor, desialylates and functionally inactivates adjacent receptors interacting with the mitogenic growth factors PDGF-BB and IGF-2. Am J Pathol. (2008) 173:1042–56. 10.2353/ajpath.2008.071081 18772331 PMC2543072

[42] Dridi L Seyrantepe V Fougerat A Pan X Bonneil E Thibault P. Positive regulation of insulin signaling by neuraminidase 1. Diabetes. (2013) 62:2338–46. 10.2337/db12-1825 23520133 PMC3712076

[43] Franceschetti S Canafoglia L. Sialidoses. Epileptic Disord. (2016) 18:89–93. 10.1684/epd.2016.0845 27621198

[44] Yang A Gyulay G Mitchell M White E Trigatti BL Igdoura SA. Hypomorphic sialidase expression decreases serum cholesterol by downregulation of VLDL production in mice. J Lipid Res. (2012) 53:2573–85. 10.1194/jlr.M027300 22984145 PMC3494259

[45] Little JP Safdar A Cermak N Tarnopolsky MA Gibala MJ. Acute endurance exercise increases the nuclear abundance of PGC-1alpha in trained human skeletal muscle. Am J Physiol Regul Integr Comp Physiol. (2010) 298:R912–7. 10.1152/ajpregu.00409.2009 20106991

[46] Rodgers JT Lerin C Haas W Gygi SP Spiegelman BM Puigserver P. Nutrient control of glucose homeostasis through a complex of PGC-1alpha and SIRT1. Nature. (2005) 434:113–8. 10.1038/nature03354 15744310

[47] Bordone L Guarente L. Calorie restriction, SIRT1 and metabolism: understanding longevity. Nat Rev Mol Cell Biol. (2005) 6:298–305. 10.1038/nrm1616 15768047

[48] Fernandez-Marcos PJ Auwerx J, Regulation Regulation of PGC-1alpha a a nodal regulator of mitochondrial biogenesis. Am J Clin Nutr. (2011) 93:884S–90. 10.3945/ajcn.110.001917 21289221 PMC3057551

[49] Planavila A Dominguez E Navarro M Vinciguerra M Iglesias R Giralt M. Dilated cardiomyopathy and mitochondrial dysfunction in Sirt1-deficient mice: a role for Sirt1-Mef2 in adult heart. J Mol Cell Cardiol. (2012) 53:521–31. 10.1016/j.yjmcc.2012.07.019 22986367

[50] Yang Y Duan W Li Y Jin Z Yan J Yu S. Novel role of silent information regulator 1 in myocardial ischemia. Circulation. (2013) 128:2232–40. 10.1161/CIRCULATIONAHA.113.002480 24218438

[51] Boengler K Lochnit G Schulz R. Mitochondria THE target of myocardial conditioning. Am J Physiol Heart Circ Physiol. (2018) 315:H1215–31. 10.1152/ajpheart.00124.2018 30004243

[52] Wallace DC. Mitochondrial diseases in man and mouse. Science. (1999) 283:1482–8. 10.1126/science.283.5407.1482 10066162

[53] Ide T Tsutsui H Hayashidani S Kang D Suematsu N Nakamura K. Mitochondrial DNA damage and dysfunction associated with oxidative stress in failing hearts after myocardial infarction. Circ Res. (2001) 88:529–35. 10.1161/01.RES.88.5.529 11249877

[54] Lin J Wu H Tarr PT Zhang CY Wu Z Boss O. Transcriptional co-activator PGC-1 alpha drives the formation of slow-twitch muscle fibres. Nature. (2002) 418:797–801. 10.1038/nature00904 12181572

[55] Wenz T Diaz F Spiegelman BM Moraes CT. Activation of the PPAR/PGC-1alpha pathway prevents a bioenergetic deficit and effectively improves a mitochondrial myopathy phenotype. Cell Metab. (2008) 8:249–56. 10.1016/j.cmet.2008.07.006 18762025 PMC2613643

[56] Atkins K Dasgupta A Chen KH Mewburn J Archer SL. The role of Drp1 adaptor proteins MiD49 and MiD51in mitochondrial fission: implications for human disease. Clin Sci. (2016) 130:1861–74. 10.1042/CS20160030 27660309

[57] Thaher O Wolf C Dey PN Pouya A Wüllner V Tenzer S., The thiol switch C684 in Mitofusin-2 mediates redox-induced alterations of mitochondrial shape and respiration. Neurochem Int. (2017) 117:167–73. 10.1016/j.neuint.2017.05.009 28527631

[58] Givvimani S Pushpakumar S Veeranki S Tyagi SC. Dysregulation of Mfn2 and Drp-1 proteins in heart failure. Can J Physiol Pharmacol. (2014) 92:583–91. 10.1139/cjpp-2014-0060 24905188 PMC4228691

[59] Li Y Wang P Wei J Fan R Zuo Y Shi M, Inhibition of Drp1 by Mdivi-1 attenuates cerebral ischemic injury *via* inhibition of the mitochondria-dependent apoptotic pathway after cardiac arrest. Neuroscience. (2015) 311:67–74. 10.1016/j.neuroscience.2015.10.020 26477985

