# Supplementary figures and images for: NEU1 Regulates Mitochondrial Energy Metabolism and Oxidative Stress Post-myocardial Infarction in Mice via the SIRT1/PGC-1 Alpha Axis
Source: Front Cardiovasc Med. 2022 Apr 25;9:821317. doi: 10.3389/fcvm.2022.821317 (PMC9081506; doi:10.3389/fcvm.2022.821317)

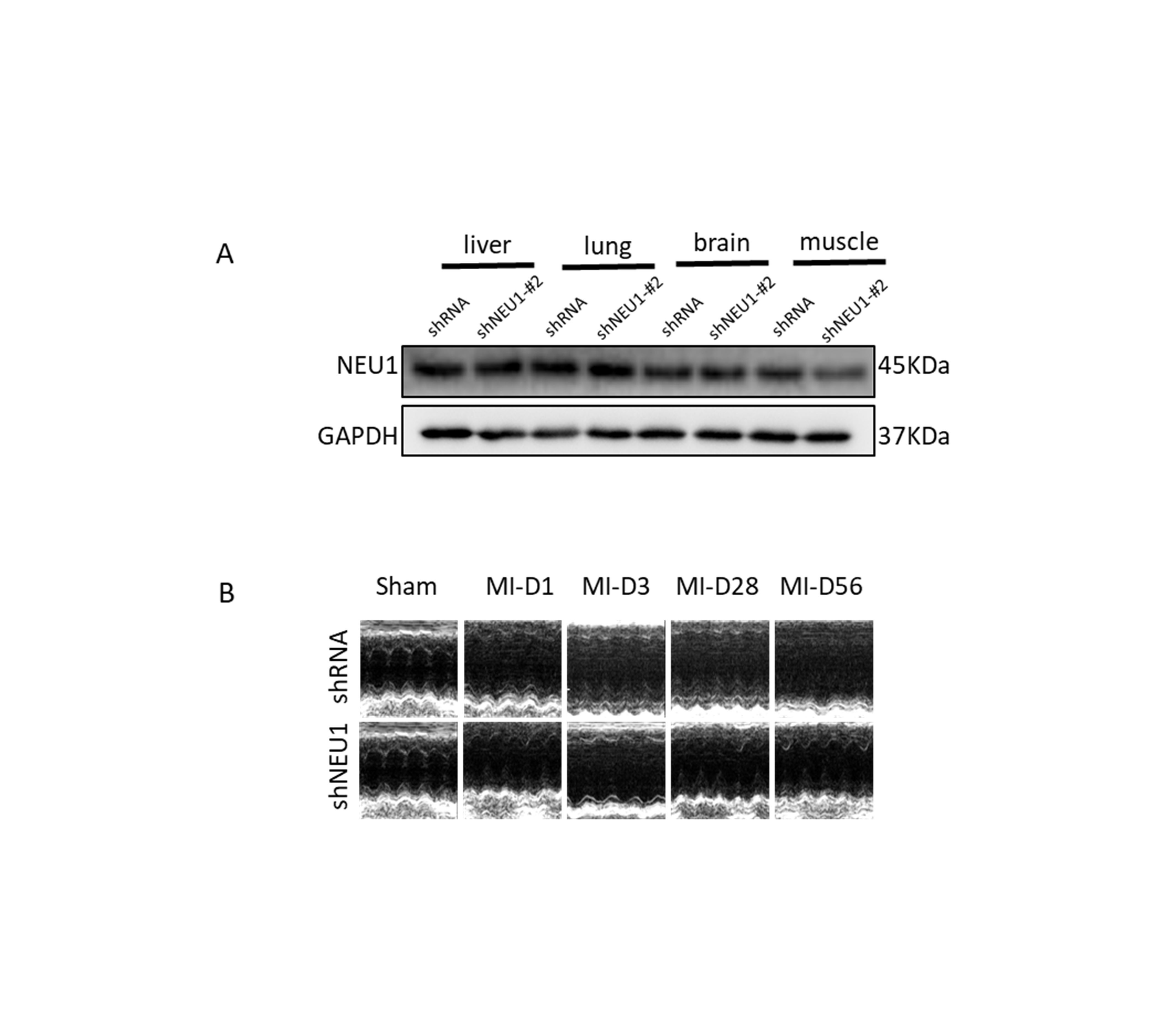

Supplement: Supplementary file 1 [file Image_1.TIF]
